# The Impacts of the COVID-19 Pandemic on Fertility Intentions of Women with Childbearing Age in China

**DOI:** 10.3390/bs12090335

**Published:** 2022-09-15

**Authors:** Tinggui Chen, Peixin Hou, Tiantian Wu, Jianjun Yang

**Affiliations:** 1 School of Statistics and Mathematics, Zhejiang Gongshang University, Hangzhou 310018, China; 2 Collaborative Innovation Center of Statistical Data Engineering Technology & Application, Zhejiang Gongshang University, Hangzhou 310018, China; 3 Department of Computer Science and Information Systems, University of North Georgia, Oakwood, GA 30566, USA

**Keywords:** fertility intentions, COVID-19, TPB, SEM

## Abstract

On 31 May 2021, the Political Bureau of the Central Committee of the Communist Party of China proposed the policy that a couple can have three children, and rolled out more supportive measures to further optimize the fertility policies. However, while the Chinese government is further optimizing its fertility policy, the sudden outbreak of COVID-19 is raging around the world, which threatens the implementation of China’s fertility optimization policy. Based on this, this paper firstly explores the impact of COVID-19 on women’s fertility intentions. Secondly, based on the Theory of Planned Behavior, this paper constructs a structural equation model to quantitatively reveal the specific factors that affect women’s fertility intentions under the epidemic, as well as their impact paths, and then puts forward corresponding suggestions for the government to solve the problem of fertility, aiming at delaying population aging and optimizing population structure. The research results show that: (1) COVID-19 lowers the fertility intentions of women of childbearing age. (2) During the pandemic, economic pressure emerged as the biggest factor affecting women’s fertility intentions. The decline in income caused by the pandemic has become an important factor in preventing women from having children. (3) The conflict between work and childbearing is still an important factor affecting the fertility intentions of women of childbearing age. The government’s provision of perfect childcare services and their strengthening of the protection of women’s employment rights and interests will greatly reduce women’s anxiety about childbearing.

## 1. Introduction

Since the 1980s, the world’s major developed countries have generally experienced reduced fertility rates, even falling below the replacement level, and long-term fertility sluggishness [1]. However, with the continuous improvement of China’s modernization level, the popularization of higher education, and the improvement of social security, first marriages and childbirth have been undertaken at later ages among young people, making China’s birth rate decline year by year. To solve the current population problems, including the continuous drop in the birth rate, the trend of delaying marriage and childbirth age, and the serious aging issue, the “separate two-child” policy (a couple in which either partner is an only child can have two children) and the “universal two-child” policy were implemented in December 2013 and January 2016, respectively. However, after the implementation of relevant policies, the problem of low fertility in China has not been solved completely, and the persistently low fertility rate has raised concerns about China’s “negative population growth”. In response to the unpopular two-child policy, on 31 May 2021, the Political Bureau of the Central Committee of the Communist Party of China proposed a policy according to which a couple can have three children, and rolled out more supportive measures to further optimize the fertility policies. This is another major innovative strategy after the two-child policy. However, right after the release of the three-child policy, COVID-19 took hold, and small-scale outbreaks of the Delta variant and the Omicron variant occurred in Xi’an, Beijing, Shanghai, Ningbo, Hangzhou, and other places. The rapid spread of the COVID-19 crisis has disrupted world trade, placed a high load on healthcare systems, and has had many negative impacts on many aspects of people’s daily lives [2]. At present, to stop the spread of the epidemic, the Chinese government is implementing multiple rounds of city closure measures, which have greatly impacted the economy and employment. Due to the experience of the lockdown and the sense of crisis in the workplace, couples of childbearing age may postpone or cancel their fertility plans [3]. Based on this, this paper explores the current status and influencing factors of fertility intentions of women of childbearing age in the epidemic context, aiming to provide a reference for the formulation of supporting measures for fertility and the balanced development of the population.

There are many studies on the impact of COVID-19 on fertility intentions, but most existing studies have focused on European and American countries affected by more severe epidemics. For example, Naya et al. [4] found that about one-third of women in the U.S. said they were delaying or hoping to have fewer children due to the COVID-19 pandemic. Luppi et al. [5] documented the impact of COVID-19 on birth planning in five European countries, and their results show that the birth plans of people surveyed in the five countries were negatively affected, but each country was affected in a different way. In addition, in terms of research scope, most studies are limited to analyzing the impact of the COVID-19 epidemic on fertility intention through single factor change. Aassve et al. [6] argued that the COVID-19 pandemic had led to a global recession, a sharp rise in unemployment and poverty among women, and a general increase in income uncertainty and unemployment levels, which made attitudes towards marriage and childbearing more negative.

Based on this, this paper constructs a structural equation model to comprehensively analyze the influences of various factors on the fertility intention of Chinese women during the epidemic period, and further proposes specific suggestions for the government to optimize fertility policies and implement fertility support measures to actively respond to the trend of population aging and expand the scale of fertility.

The paper is organized as follows: Section 2 is a literature review. Section 3 elaborates on the questionnaire design and the data collected in this paper. Section 4 conducts the empirical analysis. Section 5 concludes the whole paper and provides an outlook for future work.

## 2. Literature Review

Fertility intention is a significant factor affecting the actual fertility level of families and the total population. First, it is clear that although there is no unified standard for the concept of fertility intention and the quantification of its indicators, scholars involved in the process of discussing fertility intentions have recognized that it is not a static desire, but a tendency of individuals to have children in real life, which is constrained by real conditions in terms of the number of children, the timing of childbirth, the gender, and even the healthiness of children. For example, Wu [7] argued that fertility intention is a multi-gradient concept, which can be divided into three levels according to the closeness of its association with fertility behavior: the ideal number of children, the expected number of children, and the intended number of children. Secondly, domestic and foreign scholars have fully explored the influencing factors of fertility intention. At the macro level, social and economic factors such as education cost, housing cost and employment play an important role in influencing individual fertility intention [8]. At the same time, an increase in public expenditure by the government seeking to reduce the cost of childbirth will significantly improve the fertility intention of individuals [9]. At the micro level, support from family members will also significantly improve women’s desire to reproduce [10].

Many scholars have fully studied the changes of fertility intention during the epidemic. For instance, Druetz et al. [11] verified the negative impact of COVID-19 on fertility intention. However, most of these studies have only analyzed the impact of changes in one factor, such as the economy, on fertility intentions during the COVID-19 pandemic. First of all, the economic recession caused by COVID-19 has greatly reduced people’s fertility intention [12], with the decline in fertility being more pronounced in higher socioeconomic groups [13]. Second, the negative impact of COVID-19 on people’s mental health is also an important reason for delaying family planning [14]. Meanwhile, the impact of COVID-19 on the public health system has also affected people’s desire to have children. Damages to reproductive health services after the outbreak of COVID-19 contribute to people’s reluctance to have children [15]. People have also canceled their family plans due to concerns about the impact of COVID-19 on the health of women and fetuses [16]. In addition, the response to COVID-19 will also affect people’s desire to have children to some extent. Increased household work due to pandemic isolation has had a negative impact on family relationships. At the same time, in the case of the unbalanced division of labor in the family, an increase in housework increases the burden on women, and inhibits their fertility intention [17].

In summary, there has been a great deal of research on fertility intentions conducted by both domestic and international scholars, focusing on the concept of fertility intentions and the analysis of influencing factors. However, most studies have only explored the impact of a single factor on fertility intention during the pandemic, and few studies have comprehensively considered the impacts of various factors on fertility intention. Based on this, this paper selects women of childbearing age as the survey objects, uses questionnaire data to analyze the changes in women’s fertility willingness during the epidemic, builds a structural equation model to comprehensively analyze the influence of various factors on fertility intentions during COVID-19, explores the impact of the epidemic on their fertility willingness, and provides further information for the government. Suggestions are made to improve supporting measures for fertility, so as to meet the real needs of women in terms of fertility and increase their willingness to bear children.

## 3. Questionnaires Design and Explanation

### 3.1. Questionnaires Introduction

The questionnaire is mainly composed of three parts: deriving the basic information of the respondents, a survey on the current situation of fertility intentions, and an analysis of factors affecting fertility intentions during the epidemic.

First, referring to the research of Zhuang [18], the demographic characteristics are selected, and basic information such as age, marriage and childbirth, occupation, education, monthly income and other characteristics of the respondents are investigated.

Second, in the survey on the current situation of fertility intentions, this paper investigates the current fertility intentions of the respondents in two aspects: the expected number of children and the short-term fertility intentions. Molica et al. [14] conducted a survey of 1000 individuals in Poland whose fertility intentions were affected by the epidemic, and analyzed the changes in the fertility intentions of respondents.

Third, to quantitatively reveal the specific factors affecting women’s fertility intentions during the epidemic, the scale is divided into eight dimensions, namely, economic support, public support, policy support, family support, perceived risk, perceived behavioral control, behavioral attitudes, and fertility intentions.

A specific questionnaire can be seen in the Appendix A.

### 3.2. Description of the Questionnaire Data

A total of 443 valid questionnaires were collected online between 7 January 2022 and 21 April 2022. During the period, the more infectious COVID-19 variant strain Omicron showed an outbreak trend in Beijing, Tianjin, Shanghai, Hong Kong, Jilin, and Shaanxi, with a cumulative total of 589,870 confirmed cases (source: CDC according to Wikipedia, The New York Times), and a cumulative total of more than 890,000 people were centrally quarantined in Shanghai due to the outbreak (source: The Paper). After the release of the questionnaire, COVID-19 did not stop spreading, and appeared in Beijing and Shanghai, with 710,000 people stuck in the Shanghai containment area (source: Shanghai Health Planning Commission). Based on this, the fertility intentions of women surveyed by the questionnaire released during this period will be interpreted more diversely. Through the online platform, we collected questionnaires from all over the country. However, the number of women of childbearing age (between 22 and 46 years old) in China is about 240 million (data source: China’s sixth Census in 2010), and the sample size of this paper is 443, so there is some bias in the sample selection.

## 4. Empirical Analysis

### 4.1. Background

After the descriptive statistics of the valid questionnaires, the specific distribution of the sample is shown in Table 1. As can be seen from Table 1, the proportion of respondents in the age group of 42–46 is the smallest, the proportion of respondents in the age group 32–36 is the largest (28.25%), and the proportion of respondents in the age group 27–31 is 27.75%. In terms of educational level, the largest group is college specialists, accounting for 31.5%. The number of people with junior high school and below degrees accounted for 11%, and those with high school, secondary vocational, and secondary school degrees accounted for 14.75%. In terms of monthly gross income (before tax), the number of people with a monthly income of CNY 4500–6000 is the largest, accounting for 27.5%, while the number of people with a monthly income of CNY 6001–8000 is the second largest, accounting for 27%; the number of people with a monthly income of CNY 10,000 or more is the least, accounting for 10.5%. In terms of occupation, the number of business service personnel is the largest, accounting for 24.75%, while the number of unemployed people is the smallest, accounting for only 8%, and the remaining occupations are evenly distributed. Overall, the sample of this questionnaire is widely distributed, which is more consistent with the real situation and has a typical representation.

### 4.2. Analysis of the Current Situation of Fertility Intentions

Regarding the fertility intentions of the survey respondents, this section focuses on two perspectives: the number of expected children and short-term fertility intentions.

#### 4.2.1. The Number of Expected Children

The number of expected children as been derived via the questionnaires, and the results are shown in Table 2. The data show that the number of expected children is typically two (62.00%), one (31.44%), three or more, or another number (4.21% and 2.35% respectively). Most women of childbearing age expect to have one or two children, and only a small number of women of childbearing age expect to have three or more children or other numbers, which indicates that the number of children expected by women of childbearing age has already come to contradict the traditional concept of “more children, more happiness”.

#### 4.2.2. Short-Term Fertility Intentions

The results of the short-term fertility intentions of women of optimal age to conceive a child are shown in Table 3. As can be seen from Table 3, the short-term fertility intentions are mainly focused on the categories of “Do not think about it” (61.92%), “Do not have children” (11%), “One–two years” (10.75%), “Two–three years” (9.58%), and “Within one year” (6.75%). More than half of the women of childbearing age have no explicit intentions to have children in the short term, while the remaining women of childbearing age have predominant intentions to have children in the following 1–2 years and 2–3 years. In addition, 11% of women are sure that they have no childbearing plan within three years, which indicates that the short-term fertility intentions are low.

### 4.3. Analysis of the Change in Fertility Intentions during the Epidemic

**Figure 1:**
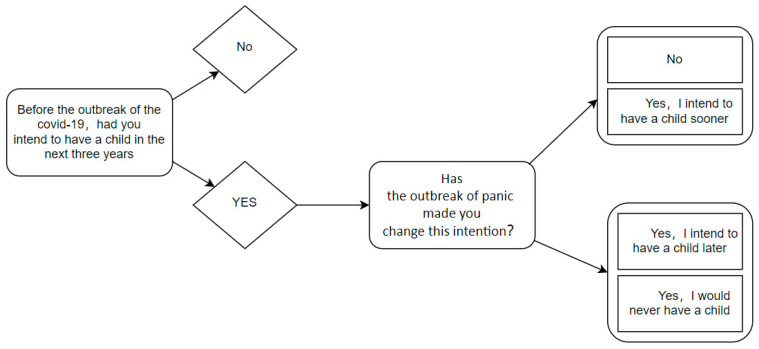
Variable interpretation plot.

The impact of COVID-19 on women’s fertility intentions is studied in this paper. Currently, the change in the fertility intentions of the female population due to the epidemic is the key variable in this questionnaire, and the variable interpretation plot is shown in Figure 1, which addresses whether female fertility intentions are affected by the COVID-19 epidemic.

**Figure 2:**
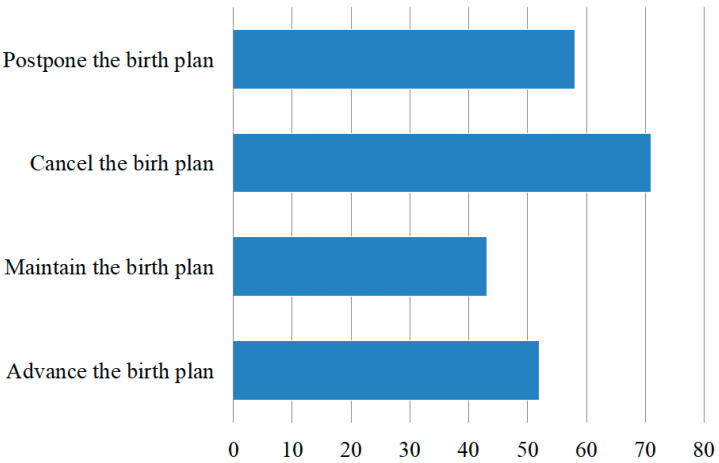
A map of changes in the fertility intentions of women of childbearing age affected by COVID-19.

As shown in Figure 2, of the 443 respondents in this questionnaire survey, 224 had a birth plan three years before the epidemic, and 219 respondents did not have a birth plan. Among the 224 respondents with fertility plans, 23% advanced their fertility plans, 19.15% maintained their fertility plans, 31.5% canceled their plans, and 26.25% decided to postpone their plans. According to the results of changes in fertility intentions, it can be seen that COVID-19 had a significant impact on the fertility intentions of the female group, in which most of the women were pessimistic about childbirth and postponed or canceled their birth plans to different degrees.

The results of the above descriptive statistical analysis show that the epidemic has negatively impacted the fertility intentions of women of childbearing age to some extent. The next section will further explore the specific factors affecting the fertility intentions of women of childbearing age, and their influence paths as affected by the epidemic.

### 4.4. A Study of Factors Influencing Women’s Fertility Intentions during the Epidemic

This section proposes research hypotheses based on the Theory of Planned Behavior and related studies, and constructs a theoretical model. In addition, it analyzes the reliability of the scale questionnaire using the SPSS26.0 software (SPSS26.0 is a professional statistical analysis software released by IBM in USA in 2019), tests the convergent validity and differential validity of the scale questionnaire using AMOS24.0 software (Amos24.0 is a structural equation modeling software released by IBM in USA), conducts a descriptive statistical analysis of the factors, and constructs a structural equation model of the factors affecting women’s fertility intentions. This section reveals the factors influencing women’s fertility intentions from a quantitative perspective and analyzes their influence paths.

#### 4.4.1. Research Hypothesis and Construction of Theoretical Model

In 1985, Ajzen added perceived behavioral control variables to the original rational behavior framework, thus forming the Theory of Planned Behavior (TPB).The TPB aims to explain human behavior rather than just predict it, and it considers that behavioral attitudes, subjective norms, and perceived behavioral control are the three prerequisites for determining behavioral intentions and behavior. The behavioral attitude refers to an individual’s positive or negative value judgment of behavior. The subjective norm refers to the social pressure individuals feel due to a particular behavior. The perceived behavioral control refers to an individual’s perception of the difficulty level of an operating behavior. Based on this, the subjective attitudes toward fertility, perceptions of family and friends’ attitudes, external social norms, and cost constraints determine the behavioral attitudes, normative beliefs, and perceived behavioral control of fertility.

**Figure 3:**
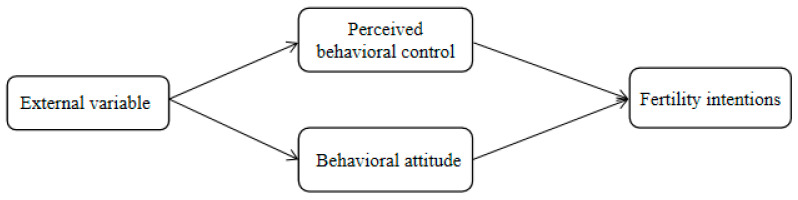
A conceptual model diagram.

Since its initial introduction, the Theory of Planned Behavior has been widely used in various industries, such as healthcare, leisure activities, travel, environmental behaviors, employment choices, shopping consumption, online activities, and online services. The predictive and explanatory power of the three variables in the model, behavior attitudes, subjective norms and perceived behavioral control, in relation to behavioral intentions has been empirically demonstrated in several studies. Ajzen et al. [19] analyzed college students’ willingness to engage in outdoor activities, and found that behavior attitude, subjective norms, and perceived behavior control all positively influenced behavior willingness, among which perceived behavior control had the greatest influence, while subjective norms had the least influence. Liao et al. [20] conducted a study of virtual banks, and found that the effect of subjective norms on use intention was not significant. Therefore, it is reasonable to construct a structural equation model of women’s fertility intentions based on TPB. However, the existing studies show that the three variables have different impacts on behavioral intentions, among which the influence of subjective norms is small, and is the weak link in the model [21]. Therefore, this paper introduces external variables, and selects perceived behavioral control and behavioral attitudes as determinants of fertility intentions; we propose research hypotheses H1 and H2 to construct a conceptual model, as shown in Figure 3.

**Hypothesis** **1** **(H1).** *Perceived behavioral control has a significant positive effect on fertility intentions.*

**Hypothesis** **2** **(H2).** *Behavioral attitude has a significant positive effect on fertility intentions.*

For the selection of external variables, this paper selected economic support, policy support, public support, family support and perceived risk as external variables for the following reasons.

Section 2 mentions that economy, employment, health, and epidemic prevention policies impact women’s fertility intentions in the epidemic context. This section unifies issues such as health factors as a variable of perceived risk. Some variables that are usually studied in demographic analyses can be used as “external” variables in social psychological research [22]. TPB distinguishes two types of external variables: First, personal background factors, including income, education, and the number of born children while deciding to have children, which may influence fertility intentions by affecting attitudes, subjective norms, and perceived behavioral control. Second, aspects of the environment, such as institutions that provide support for childcare or working parents, are described as actual controls, or actual enablers and constraints. Several studies have been conducted by national and international scholars on the external variables (namely, behavioral attitudes and perceived behavioral controls). Billari [23] showed that socioeconomic factors influenced attitudes, while economic factors and mental health influenced behavioral control. Lin et al. [24] confirmed that shared spousal responsibility and paternal care support can also enhance the control beliefs and behavioral attitudes of the population at the optimal age to conceive a child, especially women of childbearing age, and promote the formation of intentions to have another child. Zhou [25] showed that the social norms and pressures of having more children made gender discrimination in the labor market increase rather than decrease, and intensified the negative beliefs of professional women in control. Klobas [26] performed a comparative analysis of European and Asian countries, and found that young women in countries with higher levels of family- and child-friendly policy support were more likely to identify themselves with the ability to overcome fertility barriers, and their desire to give birth was higher. Yan and Zhang [27] argued that fertility policy significantly influences women’s fertility behavior through the mediator of fertility intentions. Tian [28] found via a survey of women of childbearing age in Shanghai that increasing investment in public childcare, improving the accessibility of public childcare, and providing high-quality social care support for infants and toddlers aged 0–3 years would effectively increase willingness to have children again.

**Figure 4:**
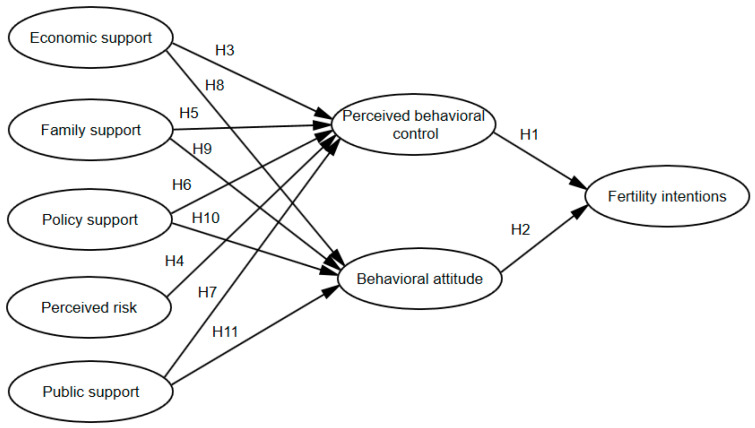
A theoretical model of fertility willingness of women of childbearing age.

Therefore, research hypotheses H3-H11 are proposed, and a theoretical model of the fertility intentions of women of childbearing age is constructed, as shown in Figure 4.

**Hypothesis** **3** **(H3).** *Economic support has a significant positive effect on perceived behavioral control.*

**Hypothesis** **4** **(H4).** *Perceived risk has a significant negative effect on perceived behavioral control.*

**Hypothesis** **5** **(H5).** *Family support has a significant positive effect on perceived behavioral control.*

**Hypothesis** **6** **(H6).** *Policy support has a significant positive effect on perceived behavioral control.*

**Hypothesis** **7** **(H7).** *Public support has a significant positive effect on perceived behavioral control.*

**Hypothesis** **8** **(H8).** *Economic support has a significant positive effect on behavioral attitudes.*

**Hypothesis** **9** **(H9).** *Family support has a significant positive effect on behavioral attitudes.*

**Hypothesis** **10** **(H10).** *Policy support has a significant positive effect on behavioral attitudes.*

**Hypothesis** **11** **(H11).** *Public support has a significant positive effect on behavioral attitudes.*

#### 4.4.2. Questionnaire Design

In this section, specific questions are designed for the eight sub-active variables of the questionnaire, and the latent variables and their codes are shown in Table 4.

#### 4.4.3. Reliability and Validity Test

(1) Reliability analysis

The reliability analysis, also called the reliability test, measures and tests the reliability and stability of the questionnaire. Cronbach’s alpha (α), which is commonly used in academia, is adopted to analyze the intrinsic reliability of the scale. Generally speaking, the closer the alpha value is to 1, the better the reliability of the questionnaire. When α is greater than 0.8, the reliability of the questionnaire is high. When α is 0.7–0.8, the reliability of the questionnaire is good. When α is 0.6–0.7, the reliability of the questionnaire is acceptable. When α is less than 0.6, some problems exist in the design or structure of the questionnaire, which should be revised.

The results of the reliability test of each variable in the questionnaire, performed by SPSS 26.0, are shown in Table 5. As can be seen from Table 5, the α coefficient of each variable is greater than 0.7, and the α coefficient of the total questionnaire reached 0.932, indicating that the questionnaire is internally consistent and stable.

(2) Validity analysis

(1) Convergence validity analysis

Convergence validity analysis is carried out by analyzing the factor loadings of each dimension. In this paper, the convergence validity of the questionnaire is determined by three indicators: standardized loading coefficient (Std.), average variance extracted (AVE), and combined reliability (CR). The questionnaire scale has convergence validity when Std. > 0.5, AVE > 0.5 and CR > 0.7. As can be seen from Table 6, the Std. values of all dimensions are greater than 0.6, which is higher than the standard value (0.5). Meanwhile, the AVE values are higher than 0.5 and the CR values are greater than 0.7, indicating that the questionnaire has good combined reliability and validity, i.e., the scale has good internal consistency and convergent validity.

(2) Distinguishing validity analysis

Distinguishing validity analysis is used to estimate the difference in the degree of correlation between a measure and different internal and external structural variables by comparing the internal correlation of the measure within a structural variable with the external correlation of each structural variable, and thus determining the distinguishing validity of the variable. If the square root of the AVE of a variable is greater than the correlation coefficient of that variable with other variables, then the distinguishing validity of that variable is good. As can be seen from Table 7, the AVE square roots of each variable (main diagonal part) are greater than the correlation coefficients of that variable and other variables, which indicates that all dimensions of the formal questionnaire have met the criteria of distinguishing validity and passed the test, and this questionnaire can thus construct structural equation models.

#### 4.4.4. Descriptive Statistics for Each Factor Score

The descriptive statistical analyses for each factor score are shown in Table 8. As can be seen from the data in the table, economic support has the highest mean score of 5.4881, and public support and perceived risk also have mean scores above 5.0, indicating that women of childbearing age are more likely influenced by these three types of factors. The mean score of policy support is lower, indicating that women of childbearing age are less influenced by macro policies at present. Meanwhile, the mean score of perceived behavioral control is 4.0994, indicating that women think they have neither enough ability to have children nor enough confidence in their fertility behavior. The behavioral attitude score is only 4.2243, indicating that women of childbearing age gradually move away from the traditional fertility concept and no longer regard fertility as a woman’s responsibility and mission, which is consistent with the findings regarding the expected number of children in the previous paper. In addition, the average score of fertility intentions is only 3.9672, indicating that women of childbearing age are less willing to have children.

In summary, the fertility intentions of women of childbearing age during the epidemic are influenced by several factors, and the specific influence paths of each factor affecting fertility intentions will be explored in the next section using structural equation modeling.

#### 4.4.5. Construction of Structural Equation Model

(1) Evaluation of the overall fitness of the structural equation model

Structural equation modeling (SEM) is an empirical analysis technique used to find the relationship between variables and verify whether the theoretical model and hypotheses are reasonable. Based on the research hypothesis and the theoretical model constructed in the previous paper, AMOS24.0 software is used to construct a structural equation model of the factors influencing women’s fertility intentions. The relative chi-square (CMIN/DF), comparative fit index (CFI), Tucker–Lewis Index (TLI), and root mean square of the error of approximation (RMSEA) are used as fitted indicators to test the fitness of the structural model. As can be seen from Table 9, the CMIN/DF value is 1.744, which is less than 3. The values of CFI, IFI, and TLI are 0.942, 0.943, and 0.934, respectively, all of which are greater than 0.9. The RMSEA value is 0.05, which is less than 0.08, i.e., all the fitted indicators are within the recommended values, indicating that the structural equation model fits better, and the theoretical model constructed in this paper is acceptable.

(2) Test results of the research hypothesis

**Figure 5:**
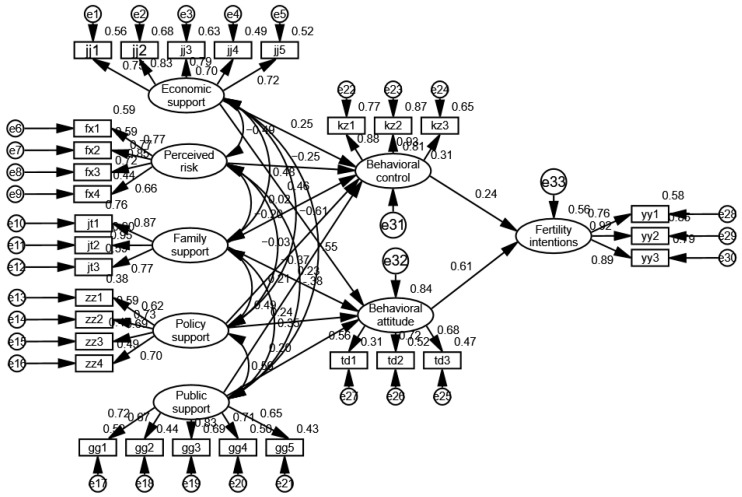
Standardized structural equation model diagram.

The results of the structural equation model of “factors influencing women’s fertility intentions”, the model relationships and standardized path coefficient estimates among the latent variables, as well as the hypothesis tests, are shown in Figure 5 and Table 10. As can be seen from Figure 5, the path coefficients of the observed variables and their latent variables in each dimension are mostly above 0.6, which indicates that the measured items of each variable are quite supportive.

As shown in Table 10, in terms of fertility intentions, the *p* values of perceived behavioral control and behavioral attitude related to fertility intentions are less than 0.001, and the standardized estimates are positive, indicating that perceived behavioral control and behavioral attitude have a significant positive effect on fertility intentions, so the original hypotheses H1 and H2 are accepted. The standardized path coefficient of behavioral attitude affecting fertility intentions is 0.611, which is significantly greater than the standardized path coefficient of perceived behavioral control (0.236), indicating that behavioral attitudes have a greater effect on fertility intentions.

In terms of perceived behavioral control, the *p* values of economic support and public support in relation to perceived behavioral control are 0.006 and 0.007, respectively, both of which are less than 0.05, and the standardized estimates are greater than 0, indicating that economic support and public support have a significant positive effect on perceived behavioral control, so the original hypotheses H3 and H7 are accepted. The *p* values of perceived risk related to perceived behavioral control are less than 0.001 and the standardized estimates are less than 0, indicating that perceived risk has a significant negative effect on perceived behavior control, so the original hypothesis H4 is accepted. Hypothesis H5 is not tested, indicating family support has no significant effect on perceived behavioral control, because most of the respondents have college and above degrees, and are more likely to have the ability to overcome fertility barriers. In addition, the positive effect of family support on perceived behavioral control may be offset by the negative effect of perceived risk. When *p* > 0.05, hypothesis H6 is not tested, indicating that there is no significant impact of policy support on perceived behavioral control because the questionnaire focuses on measures of fertility policy awareness and promotion.

In terms of behavioral attitudes, the *p* values of economic support, family support, policy support, and public support in relation to behavioral attitudes are all less than 0.05, and the standardized path coefficients are all greater than 0, indicating that economic support, family support, policy support, and public support have a significant positive effect on behavioral attitudes; therefore, the original hypotheses H8, H9, H10, and H11 are tested. The standardized path coefficient (0.463) is the largest, indicating that the economy has the greatest influence on behavioral attitudes.

#### 4.4.6. Empirical Explanation

**Figure 6:**
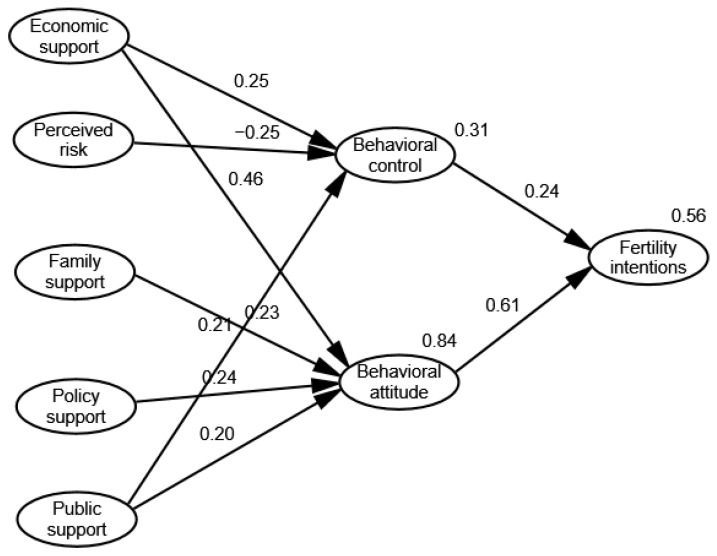
Structural equation model of fertility intentions in women of childbearing age.

Based on the Theory of Planned Behavior, this paper constructs a structural equation model of the factors influencing women’s fertility intentions, and obtains the theoretical model shown in Figure 6.

Overall, economic support and public support have a significant positive effect on perceived behavioral control. Economic support significantly impacts perceived behavioral control and public support negatively impacts perceived behavioral control, while family support and policy support do not have significant impacts on it. Economic support, family support, policy support, and public support have a significant positive effect on behavioral attitudes, in which economic support has the largest effect, followed by economic support, family support, policy support, and public support. Perceived behavioral control and behavioral attitude impact fertility intentions positively, in which behavioral attitude has the larger effect. The results are shown as follows.

(1) The role of economic support in perceived behavioral control

The standardized path coefficient of economic support affecting perceived behavioral control is 0.249, which means that if economic support increases by one standard deviation, perceived behavioral control will increase by 0.249 standard deviations. It can be seen from Figure 5 that the standardized path coefficient jj2 in the observed variables is the largest, at 0.83, indicating that increasing income has the greatest impact on perceived behavioral control, can greatly improve the confidence of women of childbearing age in reproductive behavior and their birth ability, and thus promote their fertility willingness. This conclusion is consistent with previous studies. For example, Karabchuk [29] pointed out that work and income instability were important drivers of declining birth rates.

(2) The role of perceived risk in perceived behavioral control

The standardized path coefficient of perceived risk affecting perceived behavioral control is −0.251, which means that if perceived risk increases by one unit, perceived behavioral control will be reduced by 0.251 units. Perceived risk during the epidemic contains many elements; for example, Liu et al. [30] pointed out that insufficient access to activities that fulfill fundamental needs would cause mental health issues. The perceived risks defined in this paper include the health risks, unemployment risks and psychological risks caused by the epidemic. The existence of these risks reduces women’s confidence in childbirth, and thus has a negative effect on women’s fertility intention.

(3) The role of family support in perceived behavioral control

Family support includes three parts: support from family members, family members’ situation, and family living environment. Perceived behavioral control is the degree to which individuals perceive their fertility behaviors to be controlled. Individuals consider the ability, resources, and opportunities they have to give birth, as well as the health status of the couple, job opportunities, economic situation, and the possibility that a babysitter can help them take care of the child. When there are more relevant resources and opportunities, the perceived behavioral control and fertility intentions are stronger. For example, Lin et al. [24] confirmed that family support impacts perceived behavioral controlpositively. However, family support has no significant effect on perceived behavioral control when constructing a structural equation model, because the questionnaire in this paper was released during the period when the more infectious COVID variant strain Omicron was taking hold.

During this period, respondents perceived more risks, such as health and unemployment risks. We suggest in Section 2 that the epidemic impacts women’s employment and health, while it is verified in Section 4.4.5 that perceived risk has a significant negative effect on perceived behavioral control, and the positive effect of family support on perceived behavioral control may be offset by the negative effect of perceived risk on it. In other words, respondents perceive that family support is not sufficient to equip them for childbearing and parenting during the epidemic. In addition, most of the respondents in this paper have college and above degrees, and are more likely to have the ability to overcome barriers to childbirth. Liefbroer [31] pointed out that for women, a high level of education meant a high opportunity cost of fertility, in that family and career conflicts might weaken their belief in fertility control. As such, with the development of their career, women tended to adjust their fertility intentions downward.

(4) The role of public support and policy support in perceived behavioral control

The standardized path coefficient of public support affecting perceived behavioral control is 0.211, which means that if public support increases by one unit, perceived behavioral control will increase by 0.211 units. Adequate labor rights protection and high-quality public service provision are important to easing the family–career conflicts of working women and improving their beliefs about birth control. Sound job security and social care resource support help boost the fertility intentions of women of childbearing age in China. This conclusion is the same as in the study of Tang and Li [32], which pointed out that rural–urban migrants were more vulnerable during the pandemic due to poor public support or policy support. In addition, they have faced various discriminations caused by containment interventions [33]. These may make childbearing even more difficult than for other populations. However, our analysis via structural equation modeling demonstrates that that policy support has no significant effect on perceived behavioral control. The reason for this is that the design of the measurement questions of the latent variable policy support refers to Yang [34], who focuses on whether policy awareness and support have a positive effect on perceived behavioral control, instead of focusing on specific fertility support measures, such as possible effects when increasing the construction of public child care services, which only shows that support for and knowledge of policies have no significant effect on women’s perceptions of their fertility. Therefore, it is rational to believe that policy support has no significant impact on perceived behavioral control.

(5) The role of economic support, family support, policy support and public support in behavioral attitudes

The standardized path coefficient of economic support affecting behavioral attitudes is 0.463, which means if economic support increases by one unit, behavioral attitudes will increase by 0.463 units. The standardized path coefficients of family support, policy support, and public support affecting behavioral attitudes are 0.227, 0.238, and 0.200, respectively, which are significantly smaller than 0.463, indicating that the effect of economic support on behavioral attitudes is the largest. In other words, the economic recession caused by COVID-19 has reduced their expectations and happiness regarding future births, increased their insecurity, and thus reduced their willingness to have children [35].

(6) The role of perceived behavioral control and behavioral attitudes in fertility intentions

The standardized path coefficient of perceived behavioral control affecting fertility intentions was 0.232, which means if perceived behavioral control increases by one unit, fertility intentions will increase by 0.232 units. The standardized path coefficient of behavioral attitudes affecting fertility intentions is 0.614, meaning if behavioral attitudes increase by one unit, fertility intentions will increase by 0.614 units, which is significantly greater than 0.232, indicating that behavioral attitudes have the greatest effect on fertility intentions. However, relevant studies have shown that perceived behavioral control has the greatest impact on behavioral intention [19], which is different from this conclusion. The reason for this is that the study focuses on women of childbearing age, and most respondents have two expectations regarding the number of children. In addition, Billari et al. [23] pointed out that in the case of intentions for a second child, the dominating variable for women is the one comprising positive attitudes towards a birth, while for men it is perceived control. Therefore, the result is reasonable.

## 5. Conclusions and Suggestions

This paper investigates the impact of the COVID-19 epidemic on the fertility intentions of females by distributing questionnaires, constructing structural equation models based on the Theory of Planned Behavior, using economic support, perceived risk, policy support, public support, and family support as external variables, selecting perceived behavioral control and behavioral attitude as determinants of fertility intentions, and using fertility intentions as the outcome variable to construct a structural equation model to explore female fertility intentions during the epidemic. Based on theoretical analysis and empirical research, the main findings are summarized and corresponding policy recommendations are proposed, including enhancing women’s fertility well-being and increasing fertility intentions, to achieve a fertility policy to regulate the population, delay aging in China, and optimize the population structure.

### 5.1. Conclusions

(1) Epidemic negatively affects women’s fertility intentions

After the outbreak of COVID-19, most women were pessimistic about having children, with only 27% of respondents planning to have children in the next three years. In addition, most women who had plans to have children before COVID-19 postponed or canceled their plans to varying degrees, with 31.5% of respondents planning not to have any more children and only 19.15% of respondents not changing their plans.

(2) Perceived behavioral control and behavioral attitudes positively impact fertility intentions

During the epidemic, perceived behavioral control and behavioral attitudes positively impacted women’s fertility intentions to varying degrees, with behavioral attitudes having the greatest effect. The economy has had the greatest impact on perceived behavioral control and behavioral attitude, and women’s career development and psychological factors have a significantly negative impact on perceived behavioral control, indicating that most women are still unable to solve their economic challenges, and career development and psychological problems are still important factors that hinder the female population.

### 5.2. Suggestions

(1) Provide financial support to reduce the cost of childbirth for women

According to the results of this paper, economic pressure is still one of the most important factors affecting women’s fertility intentions. As the main body of policy formulation and implementation, the government should implement a series of policies to reduce families’ economic pressures so as to improve female fertility intentions. First, granting maternity benefits for different maternity situations, such as first and second birth, can be encouraged. Secondly, improving maternity insurance policies, raising the level of maternity insurance coordination and insurance levels, and especially raising the reimbursement ratio for childbirth expenses and the level of maternity benefits, may be adopted. Third, regulating housing prices to a reasonable level, implementing preferential housing policies, providing different amounts of housing allowance for families with two and three children based on the family’s economic situation and the different circumstances of childbirth, lowering mortgage interest rates to reduce the direct cost of raising a family, and providing more financial support to women can also be implemented.

(2) Eliminate employment discrimination and alleviate women’s fertility anxiety

Female employment discrimination still exists, and the implementation of the “third child” policy will mean women encounter even more severe employment challenges. In accordance with the actual situation in each province and city, the government should further optimize the protection of female employment rights, implement maternity tax incentives to enterprises employing women who give birth, and speed up the construction of a reasonable and effective mechanism for sharing maternity costs among the state, enterprises, and families, while reducing the employment costs of enterprises and preventing them from transferring employment costs to working women, thereby solving women’s employment problems. Female employees should enjoy equal rights to men at work, and the government should improve the Labor Law and the Labor Contract Law. Companies that deprive female employees of rights and interests should be punished, and made to implement the policy thoroughly. The provisions and regulations for protecting the rights and interests of women wishing to have three children should be optimized and updated promptly to further improve the protection of women’s rights and interests.

(3) Improve public services and optimize education allocation

Most working women are struggling with balancing family and work given their limited time and energy, while the various forms of public services and infrastructure in the community do not meet their demands. Therefore, the government should adjust the construction of investment in public service resources and facilities, optimize the allocation of education, increase the construction of public childcare services, and provide financial support to improve the quality of existing childcare institutions. In addition, the government should build new public institutions and increase the number of public day childcare institutions and nurseries in places without enough childcare institutions. By providing financial subsidies to communities, the government should be able to bring the community into full play, integrate community resources, set up community-wide childcare centers (where children can be cared for by the employees of the centers), and promote diversified childcare services to meet different childcare needs.

### 5.3. Limitations and Prospects

Our study of the mechanisms affecting fertility intentions is comprehensive, and the intrinsic effects and action mechanisms are complex. This paper only selects some representative factors and indicators for analysis, without discussing in depth whether there is an indirect influence mechanism. It should subsequently look for more influencing factors, refine the content of the study, and carry out systematic research around this area in order to improve the accuracy of the research results [36]. Since there are a large number of women of childbearing age in China, and the survey volume in this paper is 443 and most of the respondents in this paper are from economically developed areas, there is a certain sample selection bias in this survey. In subsequent studies, the sample size should be expanded to avoid sample selection bias [37].

## Figures and Tables

**Table 1:** Questionnaire for survey respondents’ basic information table.

Variable	Categorize Items	Number of Persons	Percentage
Age	22–26	89	20%
	27–31	123	27.75%
	32–36	125	28.25%
	37–41	52	11.75%
	42–46	54	12.25%
Degree	Junior high school and below	48	11%
	High school, secondary school, secondary vocational,	65	14.75%
	College specialist	140	31.5%
	Bachelor’s degree	101	23%
	Graduate and above	86	19.75%
Marital status	Unmarried	89	22.25%
	Married	141	35.25%
	Remarry	110	27.5%
	Divorce	60	15%
Monthly income before tax (unit: CNY)	Under 4500	104	23.5%
	4500–6000	122	27.5%
	6001–8000	120	27%
	8001–10,000	51	11.5%
	10,000 above	46	10.5%
Occupation	Public servants	84	18.75%
	Business service personnel	110	24.75%
	Ordinary workers	100	22.75%
	Others	63	14.25%
	Farmers	51	11.5%
	Unemployed	35	8%

**Table 2:** The number of expected children.

Index	Number of Persons	Proportion (%)
One	139	31.44
Two	275	62.00
Three or more	18	4.21
Others	11	2.35

**Table 3:** Short-term fertility intentions.

Index	Number of Persons	Proportion (%)
Within one year	30	6.75
One–two years	48	10.75
Two–three years	42	9.58
Do not think about it	279	61.92
Do not have children	44	11.00

**Table 4:** List of latent variables and their codes.

Sub-Active Variables	Variable Description	Code	Measure of the Item
Economic support	Support of financial and material resources for fertility behavior	jj1	Increase medical care reimbursement rates
		jj2	Income
		jj3	Increase the housing provident fund
		jj4	Increase pension
		jj5	Maternity subsidies
Policy support	Support of fertility policy for fertility behavior	zc1	The influence of the fertility policy
		zc2	Knowledge of the fertility policy
		zc3	Support degree of the fertility policy
		zc4	The promotion degree of the fertility policy
Public support	Support of social public services for fertility behavior	gg1	Increase the number of years of compulsory education
		gg2	Improve medical services
		gg3	Increase childcare institutions
		gg4	Increase the number of after-school tutoring hours
		gg5	Increase pension institutions
Family support	Support of family for fertility behavior	jt1	The degree of family support
		jt2	Situation of the family members
		jt3	Family living environment
Perceived risk	Perception of the psychological, health, and other risks of fertility	fx1	Health risk
		fx2	Risk of unemployment
		fx3	Financial burden
		fx4	Psychological anxiety
Perceived behavioral control	Fertility intention in the short term (within three years)	kz1	Time and energy
		kz2	Economic capability
		kz3	Confidence
Behavioral attitude	Perceptions of fertility and cost constraints	td1	Responsibility and mission
		td2	Wise behavior
		td3	It is worth promoting
Fertility intentions	Positive or negative value evaluation of fertility behavior	yy1	Be willing to have children
		yy2	Plan to have children in the next three years
		yy3	Encourage relatives and friends to have children in the next three years

**Table 5:** Results of the reliability analysis.

Variable	The Number of Terms	α
Economic support	5	0.871
Public support	5	0.839
Perceived risk	4	0.843
Policy support	4	0.776
Family support	3	0.894
Perceived behavioral control	3	0.905
Behavioral attitude	3	0.724
Fertility intentions	3	0.888
Totality	30	0.932

**Table 6:** Convergence validity test table.

Dimension	Question Item	UnStd.	S.E.	t-Value	*p*	Std.	SMC	CR	AVE
Economic support	jj1	1.000				0.751	0.564	0.872	0.578
	jj2	1.059	0.075	14.165	***	0.827	0.684		
	jj3	1.016	0.075	13.553	***	0.793	0.629		
	jj4	0.893	0.075	11.909	***	0.703	0.494		
	jj5	0.834	0.068	12.231	***	0.721	0.520		
Perceived risk	fx1	1.000				0.765	0.585	0.848	0.585
	fx2	0.994	0.077	12.878	***	0.768	0.590		
	fx3	1.119	0.08	14.027	***	0.850	0.723		
	fx4	0.933	0.085	11.034	***	0.664	0.441		
Family support	jt1	1.000				0.871	0.759	0.900	0.752
	jt2	1.057	0.049	21.492	***	0.950	0.903		
	jt3	0.877	0.054	16.295	***	0.770	0.593		
Policy support	zz1	1.000				0.803	0.645	0.842	0.573
	zz2	1.020	0.077	13.266	***	0.798	0.637		
	zz3	0.819	0.070	11.751	***	0.701	0.491		
	zz4	0.867	0.072	12.086	***	0.720	0.518		
Public support	gg1	1.000				0.720	0.518	0.841	0.517
	gg5	1.057	0.102	10.327	***	0.652	0.425		
	gg2	0.960	0.091	10.550	***	0.667	0.445		
	gg3	1.244	0.097	12.855	***	0.831	0.691		
	gg4	1.080	0.096	11.204	***	0.710	0.504		
Perceived behavioral control	kz1	1.000				0.878	0.771	0.906	0.764
	kz2	1.089	0.051	21.562	***	0.933	0.870		
	kz3	0.928	0.053	17.618	***	0.807	0.651		
Behavioral attitude	td1	1.000				0.776	0.602	0.759	0.513
	td2	0.764	0.088	8.700	***	0.665	0.442		
	td3	0.962	0.109	8.793	***	0.703	0.494		
Fertility intentions	yy1	1.000				0.763	0.582	0.894	0.738
	yy2	1.182	0.071	16.578	***	0.920	0.846		
	yy3	1.171	0.073	16.115	***	0.887	0.787		

Note: *** means it is outstanding at the 0.1% level.

**Table 7:** Distinguishing validity tests.

	AVE	D	A	C	B	L	F	R	E
Fertility intentions(D)	0.738	0.859							
Behavioral attitude (A)	0.513	0.613	0.716						
Perceived behavioral control (C)	0.764	0.500	0.383	0.874					
Public support (B)	0.517	0.476	0.651	0.433	0.719				
Policy support (L)	0.573	0.581	0.692	0.344	0.559	0.757			
Family support (F)	0.752	0.526	0.574	0.241	0.346	0.491	0.867		
Perceived risk (R)	0.585	0.390	0.442	0.444	0.374	0.37	0.195	0.765	
Economic support (E)	0.578	0.648	0.704	0.480	0.546	0.61	0.481	0.486	0.76

**Table 8:** Comprehensive score table for each variable.

Variable	Number of Cases	Minimum	Maximum	Average	Standard Deviation
Economic support	443	1	7	5.4881	1.1301
Public support	443	1	7	5.4079	1.1179
Perceived risk	443	1	7	5.03	1.3515
Family support	443	1	7	4.6881	1.2634
Policy support	443	1	7	4.2458	1.1813
Perceived behavioral control	443	1	7	4.0994	1.5727
Behavioral attitude	443	1	7	4.2243	1.1135
Fertility intentions	443	1	7	3.9672	1.1226

**Table 9:** Results of the model fitting.

Adaptation Index	CMIN/DF	GFI	AGFI	CFI	IFI	TLI	RMSEA
Recommended value	<3	>0.8	>0.8	>0.9	>0.9	>0.9	<0.08
Fitted value	1.744	0.87	0.843	0.942	0.943	0.934	0.05

**Table 10:** Results of the hypothesis test.

Assume	UnStd.	S.E.	C.R.	*p*	Std.	Conclusion
H1	0.150	0.036	4.220	***	0.236	Support
H2	0.646	0.079	8.152	***	0.611	Support
H3	0.381	0.137	2.774	0.006	0.249	Support
H4	−0.338	0.094	−3.608	***	−0.251	Support
H5	0.022	0.088	0.251	0.802	0.017	Nonsupport
H6	−0.059	0.172	−0.343	0.731	−0.032	Nonsupport
H7	0.387	0.144	2.682	0.007	0.211	Support
H8	0.426	0.071	6.014	***	0.463	Support
H9	0.179	0.045	4.000	***	0.227	Support
H10	0.267	0.089	3.003	0.003	0.238	Support
H11	0.221	0.072	3.060	0.002	0.200	Support

Note: *** means it is outstanding at the 0.1% level.

## Data Availability

The data used to support the findings of this study are available from the first author upon request.

## Appendix A

**Questionnaire on influencing factors of reproductive intention among women of childbearing age during COVID-19**

Dear Madam:

Hello! Thank you for taking time out of your busy schedule to accept this survey. This survey aims to study the impact of COVID-19 on Chinese reproductive-age women’s fertility intentions. This survey is conducted in an anonymous way, and the survey results are for academic research only. Personal information involved will be kept strictly confidential so you are expected to answer according to the actual situation. Thank you very much for your understanding and cooperation. Wish you a happy life!

(One) Personal information

1. How old are you?

A. 22–26

B. 27–31

C. 32–36

D. 37–41

E. 42–46

2. What is your degree?

A. Junior high school and below

B. High school, Secondary vocational, Secondary school

C. College Specialist

D. Bachelor’s degree

E. Graduate and above

3. What is your current marital status?

A. Unmarried

B. Married

C. Remarry

D. Divorce

4. What is your monthly income before taxes?

A. Under 4500

B. 4500–6000

C. 6001–8000

D. 8001–10,000

E. 10,000 above

5. What is your occupation?

A. Public servants

B. Business service personnel

C. Ordinary workers

D. Others

E. Farmers

F. Unemployed

(Two) Survey of the current situation of fertility intentions

1. If you don’t consider fertility policies and other conditions, you think that the average family has the most ideal to have several children?

A. 0

B. 1

C. 2

D. 3 and more than 3

2. How soon do you plan to have children?

A. Within one year

B. One–two years

C. Two–three years

D. Didn’t think about it

D. Do not have children

3. Before the outbreak of COVID-19, had you intend to have a child in the next three years?

A. Yes

B. No

4. Has the outbreak of the epidemic changed your fertility intentions?

A. Yes, I’m planning to have a child sooner.

B. Yes, I’m going to postpone my birth plan.

C. Yes, I don’t plan to have any more babies.

D. No, it did not change my fertility intentions.

(Three) Investigation on the influencing factors of fertility willingness under the epidemic

Please tick the most appropriate option based on your own judgment and ideas.

**Topic\Options**	**Strongly Disagree**	**Disagree**	**Somewhat Disagree**	**Moderately**	**Somewhat Agree**	**Agree**	**Strongly Agree**
I think income has a big impact on fertility intention.							
I think an increase in medical reimbursement will increase my fertility intention.							
I think increasing the housing provident fund will increase my fertility intention.							
I think the pension hike will boost my fertility intention.							
I think that giving out maternity subsidies will increase my fertility intention.							
I think fertility policy will affect my fertility intention.							
I think the awareness of fertility policy will affect the fertility intention.							
I support the government’s introduction of corresponding birth control measures.							
I think the government’s promotion of the three-child policy has increased my fertility intention.							
I think the increase in the number of years of compulsory education will promote my fertility intention.							
I think improved medical services will boost my fertility intentions.							
I think an increase in childcare will increase my fertility intention.							
I think the school’s increased after-school tutoring hours will improve my fertility intention.							
I think the increase in nursing homes will boost my fertility intention.							
I think the level of support from family members affects my fertility intention.							
I think family members’ situation can influence my fertility intention.							
I think family life circumstances can affect my fertility intention.							
I think the health risks brought about by the epidemic have a great impact on fertility intention.							
I think the unemployment risk brought about by the epidemic have a great impact on fertility intention.							
I think women’s career development risks have a big impact on fertility intention.							
I think having children will increase my financial burden.							
I think the psychological anxiety brought about by the epidemic have a great impact on fertility intentions.							
I think I have enough energy and time to raise children.							
I think I have enough confidence in raising children.							
I think I am financially capable enough to raise a child.							
I think fertility is a responsibility and a mission.							
I think reproductive behavior is sensible.							
I think the three-child policy is worth promoting.							
I would like to have children.							
I plan to have children within the next three years.							
I would like to persuade my relatives and friends to have children within the next three years.							
